# MHC Class II Molecules Enhance Toll-Like Receptor Mediated Innate Immune Responses

**DOI:** 10.1371/journal.pone.0008808

**Published:** 2010-01-20

**Authors:** Remo Frei, Johanna Steinle, Thomas Birchler, Susanne Loeliger, Caroline Roduit, Dirk Steinhoff, Reinhart Seibl, Katja Büchner, Reinhard Seger, Walter Reith, Roger P. Lauener

**Affiliations:** 1 Division of Immunology/Allergology, University of Zurich, Children's Hospital, Zurich, Switzerland; 2 Section of Clinical Immunology, University Hospital Zurich, Zurich, Switzerland; 3 Department of Pathology and Immunology, University of Geneva Medical School, Geneva, Switzerland; Massachusetts General Hospital/Harvard University, United States of America

## Abstract

**Background:**

Major histocompatibility complex (MHC) class II molecules play crucial roles in immune activation by presenting foreign peptides to antigen-specific T helper cells and thereby inducing adaptive immune responses. Although adaptive immunity is a highly effective defense system, it takes several days to become fully operational and needs to be triggered by danger-signals generated during the preceding innate immune response. Here we show that MHC class II molecules synergize with Toll-like receptor (TLR) 2 and TLR4 in inducing an innate immune response.

**Methodology/Principal Findings:**

We found that co-expression of MHC class II molecules and TLR2 or TLR4 in human embryonic kidney (HEK) cells 293 leads to enhanced production of the anti-microbial peptide human-β-defensin (hBD) 2 after treatment with TLR2 stimulus bacterial lipoprotein (BLP) or TLR4 ligand lipopolysaccharide (LPS), respectively. Furthermore, we found that peritoneal macrophages of MHC class II knock-out mice show a decreased responsiveness to TLR2 and TLR4 stimuli compared to macrophages of wild-type mice. Finally, we show that MHC class II molecules are physically and functionally associated with TLR2 in lipid raft domains of the cell membrane.

**Conclusions/Significance:**

These results demonstrate that MHC class II molecules are, in addition to their central role in adaptive immunity, also implicated in generating optimal innate immune responses.

## Introduction

Innate immunity mediates the first immune response to evolutionary conserved foreign patterns upon their recognition by pattern recognition receptors. TLR are the principal pattern recognition receptors. They recognize conserved molecular patterns of microbes, initiate rapid anti-microbial responses protecting the host during the fist days of infection, and generate danger-signals including cytokines and co-stimulatory molecules required for activation of the adaptive immune system [1]. Regulation of the TLR signaling cascade is crucial for adequate inflammatory responses and innate host defense, but also for adaptive immune responses [2], [3]. Additional signals for T helper cell activation are provided by MHC class II molecules presenting processed foreign peptides to antigen-specific T helper cells.

Patients suffering form septic shock express less MHC class II molecules and show reduced LPS responsiveness indicating a role of MHC class II molecules in innate immunity [4]. Here we describe a function of MHC class II molecules in the innate host defence. Expression of MHC class II molecules enhanced the responsiveness of TLR to their ligands through co-localization in lipid raft domains of the membrane.

## Results

### HLA-DR Increases the Responsiveness in Plasma Membrane-Bound TLR Expressing HEK293 Cells

To assess a potential role of MHC class II molecules in the innate immune response we compared expression of hBD-2 or TNF in HEK293 cells transfected with HLA-DR and control cells. The HEK293 cell line expresses neither HLA-DR molecules nor TLR2, TLR4, or TLR7. We stably transfected these cells with the respective TLR and/or HLA-DR. Quantitative real-time PCR showed that TLR2 expression levels were 1.8 times higher in cells expressing only TLR2 compared to cells expressing HLA-DR and TLR2, while the HLA-DR/TLR4 or HLA-DR/TLR7 positive cells expressed more TLR then the cells positive for only TLR4 or TLR7 (3.5 times for TLR4 and 2.8 times for TLR7) (Figure S1A). Since wild-type HEK293 cell lines and HEK293 cell line expressing HLA-DR were of different origin, we compared the gene expression of relevant molecules of the TLR signaling cascade (Figure S1B). We did not detect significant differences in the gene expression of these molecules.

**Figure 1:**
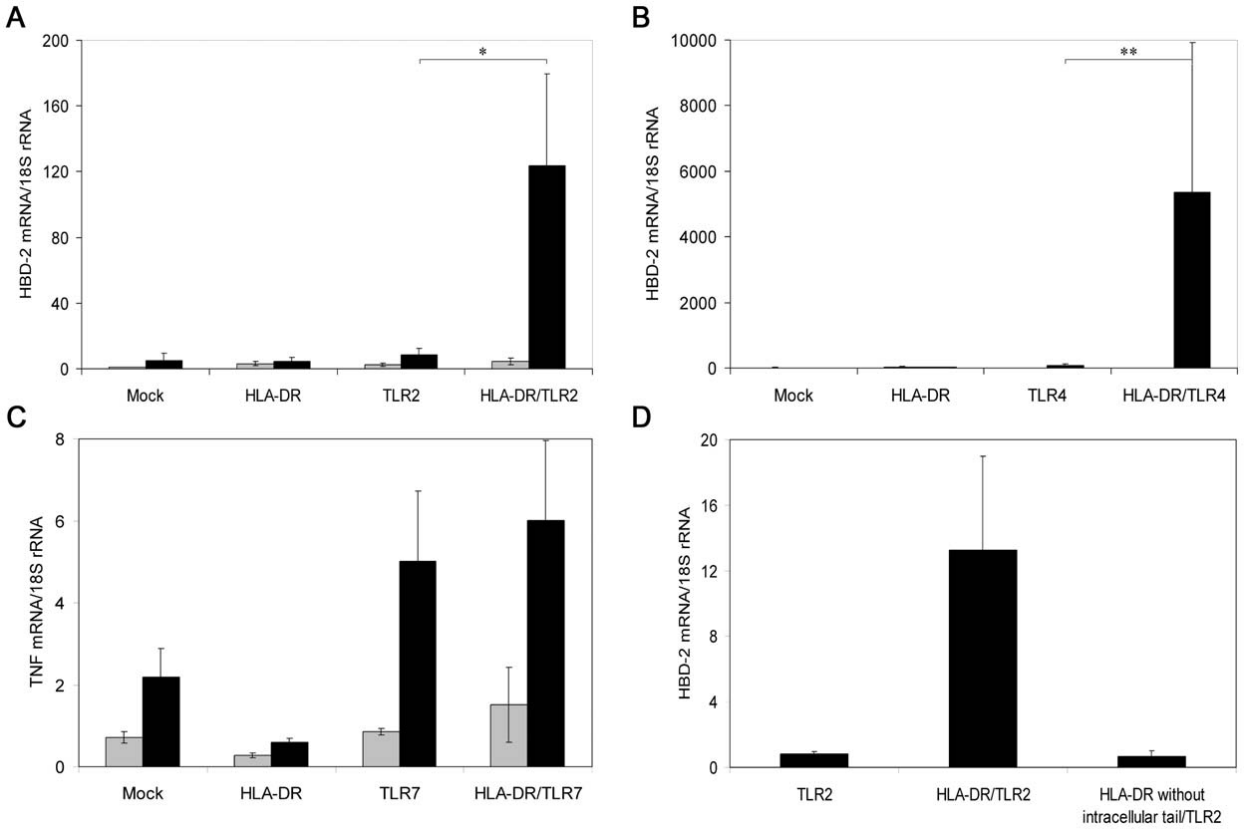
Expression of HLA-DR enhances TLR2 and TLR4 dependent activation of HEK293 cells. (A, B) Co-expression of HLA-DR in TLR2^+^ or TLR4^+^ HEK293 cells leads to enhanced hBD-2 expression (n≥3) after stimulation with BLP and LPS, respectively (black bars) compared to untreated cells (grey bars). (C) Co-expression of HLA-DR in TLR7^+^ cells did not increase the TNF expression (n = 3) after treatment with Loxoribine (black bars) compared to untreated cells (grey bars). (D) Co-expression of HLA-DR lacking the intracellular part in TLR2^+^ HEK293 cells failed to enhance hBD-2 expression after stimulation with BLP (n = 3). Error bars represent standard errors; * p<0.05, ** p = 0.055.

To investigate the responsiveness of the HEK293 cell variants, we stimulated the cells with TLR2 ligand BLP, TLR4 ligand LPS, or TLR7 ligand Loxoribine and assessed the expression of the hBD-2 gene (TLR2 and TLR4) and of the TNF gene (TLR7) by quantitative real-time PCR (Figure 1). In agreement with previous reports [5], [6], expression of TLR2 or TLR4 alone led to an increase in hBD-2 production (3.7 times for TLR2, 31 times for TLR4) and expression of TLR7 increased TNF production (5.9 times) after stimulation. Expression of HLA-DR alone was not sufficient to confer responsiveness to BLP, LPS, or Loxoribine. Co-expression of HLA-DR and TLR2 or TLR4 markedly enhanced production of the antimicrobial peptide compared to cells positive for TLR2 and TLR4 only (14.4 times for TLR2 and 75.1 times for TLR4, respectively). Co-expression of HLA-DR and TLR7 failed to increase the TNF response compared to TLR7 positive cells. Further, co-expression of HLA-DR lacking the intracellular part failed to enhance the BLP-induced hBD-2 gene expression of HEK293 cells positive for TLR2 compared to cells positive for only TLR2 (Figure 1D).

### Peritoneal Macrophages of MHC Class II Knock-Out Mice Show Impaired Responsiveness

**Figure 2:**
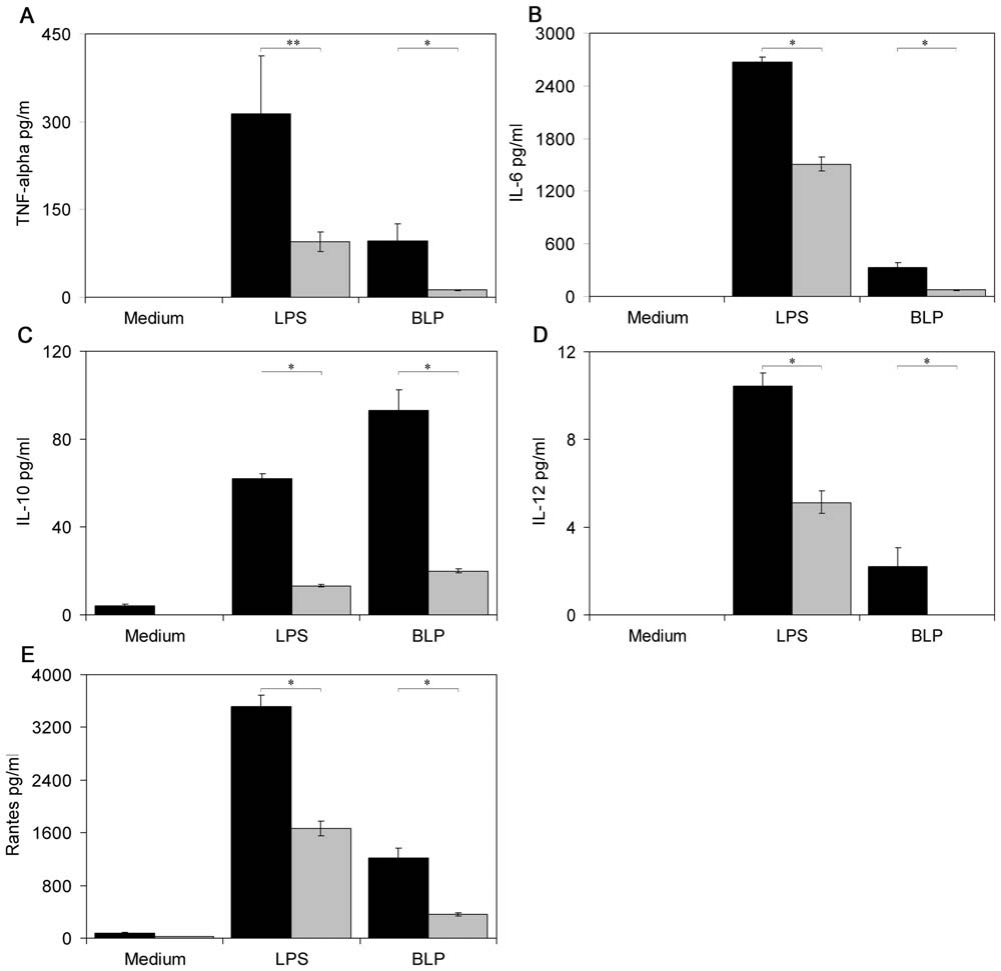
Peritoneal macrophages from MHC class II knock-out mice show impaired responsiveness to BLP and LPS. (A–E) Cytokine secretion of peritoneal macrophages from MHC class II knock-out mice (grey bars; n≥4) is reduced compared to wild-type control mice (black bars; n≥3) after *ex vivo* stimulation with BLP and LPS. Error bars represent standard errors; ** p = 0.053, * p<0.05.

We evaluated the contribution of MHC class II molecules to the innate immune response in another experimental model. We compared the cytokine response of peritoneal macrophages isolated from wild-type and MHC class II knock-out mice [7] of the same genetic background (C57BL/6) following treatment with BLP or LPS. We found reduced TNF-α, IL-6, IL-10, IL-12, and RANTES secretion in peritoneal macrophages of MHC class II knock-out mice compared to those of the wild-type mice (Figure 2). The gene expression of TLR2, TLR4, CD14, and of some relevant molecules of the TLR signaling cascade were comparable in wild-type and MHC class II knock-out mice (Figure S2).

### TLR2 and HLA-DR Are Able to Interact Physically

**Figure 3:**
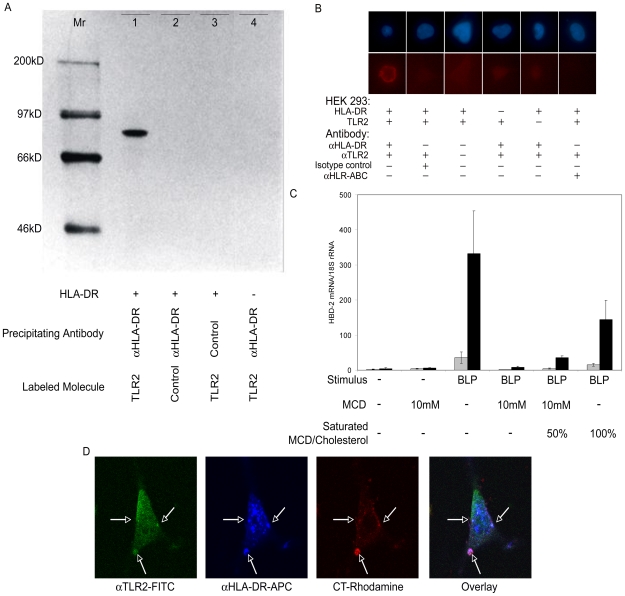
TLR2 and HLA-DR interact physically in lipid raft domains. (A) Co-precipitation of labeled recombinant TLR2 (lane 1, 3, 4) or a control protein (lane 2, luciferase) with HLA-DR molecules purified by immunoprecipitation with anti HLA-DR antibodies (lane 1, 2, 4) or control antibodies (lane 3) from cell lysates of HLA-DR positive (lane 1, 2, 3) or negative (lane 4) HEK293 cells. A ^14^C-labeled molecular weight marker (in kD) is shown in the left lane. (B) Proximity ligation assays showed that TLR2 and HLA-DR are close together (red signal), while TLR2 and HLA-ABC do not co-localize (no red signal). Nuclei are stained by Hoechst 33342 (blue). (C) Treatment of TLR2^+^ (grey bars; n = 3) or HLA-DR^+^/TLR2^+^ (black bars; n = 3) HEK293 cells with MCD, a lipid raft-destroying agent, prior to stimulation with BLP leads to impaired hBD-2 gene expression. The effect is restored via adding MCD saturated with cholesterol acting as cholesterol donor. Error bars represent standard errors. (D) Immunofluorescence microscopy showed that TLR2 (αTLR2-FITC; green) and HLA-DR (αHLA-DR-APC-Cy9; blue) co-localize in lipid raft domains (CT-rhodamine; red) of HLA-DR^+^/TLR2^+^ HEK293 cells (indicated by the white arrows). Pictures are representatives of at least five experiments.

We then assessed whether the functional synergism between TLR and MHC class II molecules might be paralleled by their physical interaction. Because the enhanced responsiveness in TLR2 expressing cells was more significant than in TLR4 expressing cells, we carried out co-precipitation experiments with the HEK293 cell line expressing TLR2 and HLA-DR. We found that recombinant radioactively labeled TLR2 can be co-precipitated with HLA-DR (Figure 3A). Furthermore, proximity ligation assay (PLA) [8] showed that TLR2 was located closely together with HLA-DR on HEK293 cells, whereas we did not detect a co-localization of TLR2 and HLA-ABC (Figure 3B).

### TLR2 and HLA-DR Collaborate in Aggregated Plasma Membrane Rafts

We were not able to inhibit the enhanced responsiveness of HLA-DR^+^/TLR2^+^ HEK293 cells with antibodies directed against HLA-DR (data not shown). Moreover, the responsiveness of intracellular located TLR7 was not enhanced by HLA-DR co-expression. So, we wondered whether MHC class II molecules and TLR2 collaborate in lipid raft domains of the cell membrane. These membrane domains are rich in cholesterol and essential for efficient signaling processes [9], [10], [11], [12]. A role of lipid rafts in TLR-signaling has previously been reported [13]. We found that Methyl-β-cyclodextrin (MCD) mediated destruction of lipid raft domains inhibited BLP-triggered expression of the hBD-2 gene in HLA-DR^+^/TLR2^+^ cells (Figure 3C). Adding of MCD saturated with cholesterol acting as cholesterol donor [14] restored the BLP-mediated responsiveness of HLA-DR^+^/TLR2^+^ cells (Figure 3C). Moreover, immunofluorescence microscopy revealed that HLA-DR and TLR2 co-localize in aggregated plasma membrane rafts (Figure 3D).

## Discussion

Our results reveal a novel and unexpected function of MHC class II molecules. We show here that MHC class II molecules can play a key role in innate immunity by enhancing TLR-mediated cellular activation and antimicrobial effector mechanisms. We suggest an underlying mechanism enhancing TLR-signaling through co-localization with MHC class II in aggregated plasma membrane rafts rather than direct binding of the ligand to MHC class II. This is in agreement with the finding that MHC class II failed to enhance the responsiveness of intracellularly located TLR7. The observed effect is dependent on the intracellular part of MHC class II although this part is not crucial for the localization of MHC class II in aggregated plasma membrane rafts [15].

The induction of MHC class II molecule expression by TLR agonists [16], [17], thus, not only enables effective antigen presentation for the activation of adaptive immunity, but also functions as a positive feedback mechanism enhancing TLR-mediated responses. The innate immune response is a tightly regulated process involving numerous stimulatory and inhibitory molecules [18]. Over-reaction of the innate immune system may lead to chronic inflammation, allergy, and autoimmunity. Insufficient activation results in both inadequate protection during the first days of infection and in inefficient or inappropriate activation of the adaptive immune system, potentially leading to disease or death. We have identified MHC class II molecules as novel participants in the mechanisms controlling innate immune responses.

## Materials and Methods

### Cell Lines and Media

The HEK 293 cell line was obtained from the American Type Collection Culture. HEK293 cells transfected with HLA-DR (α-, β-, and invariant chain) were a gift from Dr. J. Neefjes (Netherlands Cancer Institute). These cells were stably transfected with an empty pCEP4 plasmid (Invitrogen), a pUNO-hTLR2/4/7 plasmid (InvivoGen), or with three plasmids coding for each chain of HLA-DR lacking the intracellular tail using the TransIT-293 Transfection Reagents (Mirus). Cells were grown in DMEM (Gibco BRL) supplemented with 10% low endotoxin FBS (Hyclone) and 1% of an antibiotic-antimycotic solution (Gibco BRL). Depending on the resistance genes present, medium was supplemented with additional selection antibiotics G418, Hygromycin (Roche Diagnostics), Ouabain (Sigma), or Blasticidin (InvivoGen).

### Cell Stimulation, Modulation of Cellular Cholesterol, and Total RNA Extraction

HEK293 cells were grown in 6-well plates at 37°C in 5% CO_2_ until they were 80% confluent and then stimulated for 6 h with BLP (1 µg/ml, Pam3Cys-SKKKK, EMC microcollections), LPS (1 µg/ml, List Biological Laboratories) or Loxoribine (1 mM, InvivoGen). For modulation of cellular cholesterol, the cells were treated before stimulation with BLP for 1 h with MCD (10 mM, Sigma-Aldrich), a 1∶1 mixture of free MCD and MCD saturated with cholesterol (Sigma-Aldrich), or MCD saturated with cholesterol alone. MCD saturated with cholesterol was prepared as described elsewhere [14]. In brief, 5 mM MCD solution in DMEM medium was added to dry cholesterol. The mixture was vortexed, sonicated, and shaken overnight at 37°C. The QIAmp RNA Blood Mini Kit (Qiagen) supplemented with RNase-free DNase (Qiagen) was used for total RNA isolation.

### Ex Vivo Mouse Experiments

All mice were kept either at the Animal Facilities of the University of Geneva Medical School or at the Section of Clinical Immunology, University Hospital Zurich under optimised hygienic conditions. All experiments were conducted in accordance with the ethical guidelines of the Animal Studies Ethics Committee. Peritoneal macrophages were isolated from wild-type (Harlan) and MHC class II deficient [7] C57BL/6 mice. The macrophages were cultured in X-VIVO 15 (BioWhittaker) over night and stimulated (BLP 10 µg/ml; LPS 10 ng/ml) for 4 h in DMEM supplemented with 10% low endotoxin FBS and 1% of an antibiotic-antimycotic solution. Supernatants were analyzed with the Bio-Plex HTF-System (Bio-Rad) using a kit of Bio-Rad.

### Quantitative Real-Time PCR

Reverse transcription and quantitative real-time PCR were performed with reagents from Applied Biosystems according to the manufacturer's instructions. Quantitative real-time PCR was performed with an ABI Prism 7700 Sequence Detection System™ (Applied Biosystems). For hBD-2 mRNA, primer sequences were 5′-GAGGAGGCCAAGAAGCTGC-3′ (300 nM)and 5′-CGCACGTCTCTGATGAGGG-3′ (300 nM), and the probe had the sequence 5′-TGGCTGATGCGGATTCAGAAAGGG-3′ (250 nM). For 18S rRNA primer sequences were 5′-AGTCCCTGCCCTTTGTACACA-3′ (200 nM) and 5′-GATCCGAGGGCCTCACTAAAC-3′ (200 nM), and the probe had the sequence 5′-CGCCCGTCGCTACTACCGATTGG-3′ (250 nM). All oligonucleotides were synthesized by Microsynth. All other primers and probe sets were purchased as pre-developed assays by Applied Biosystems. Results were normalized relative to 18S rRNA using the comparative (ΔΔCt) method according to the manufacturer's instructions (Applied Biosystems).

### Co-Immunoprecipitations

Immunoprecipitations were performed using the ‘Cellular Labeling and Immuoprecipitation Kit’ according to the manufacturer's instructions (Roche Diagnostics). HEK293 cells were collected in lysis buffer, sonificated, and insoluble material was removed by centrifugation. To eliminate non-specific binding, supernatants were incubated with 50 µl protein A agarose and cleared by centrifugation. Immunoprecipitation was then performed by mixing 1 µg of unlabeled anti-HLA-DR antibodies with 40 µl of the pre-cleared supernatant and 50 µl protein A-agarose. The immunoprecipitates were collected by centrifugation, washed and dissolved in 50 mM TBS (pH 7.5). Recombinant radio-labeled proteins were synthesized using the TNT T7 Quick Coupled Transcription/Translation System (Promega) and pBluescript KS (−) plasmids expressing TLR2 or luciferase. The recombinant molecules were added to the immunoprecipitated samples and complexes were collected by centrifugation, washed, and analyzed by SDS-PAGE.

### Proximity Ligation Assay (PLA)

Proximity ligation assays [8] were performed with the Duolink in situ PLA kit of Olink Bioscience using a slightly different protocol. Paraformaldehyde fixed HEK293 cells expressing HLA-DR, TLR2, or both molecules were incubated with primary antibodies made either in mouse or goat directed against HLA-DR (BD Bioscience), TLR2 (R&D Systems), HLA-ABC (BD Pharmingen), or an isotype control. Secondary anti-mouse and anti-goat antibodies, conjugated to PLA oligonucleotides probe, were used to detect primary antibodies. If these probes are located close enough together, they hybridize and are ligated. Ligated probes were amplified by a rolling-circle PCR, detected with fluorescently labeled probes, and visualized with fluorescence microscopy. Between all steps, cells were washed with PBS.

### Immunofluorescence Microscopy

HEK293 cells expressing HLA-DR and TLR2 were grown overnight on coverslips and fixed for 15 min. with 3% paraformaldehyde. Cells were stained by sequential 45 min. incubations with rhodamin-labeled cholera-toxin-β-subunit (0.5 µg/ml; List Biological Laboratories) to visualize the lipid raft domains [9], a FITC anti-human TLR2 monoclonal antibody (T2.1; eBioscience), and an APC-Cy9 anti-human HLA-DR monoclonal antibody (L243; BD Biosciences). Cells were washed three times with PBS between each staining step. Cells were mounted in Glycergel (DakoCytomation) and observed with a Leica TCS SL confocal microscope using the 63x/1.2 W CORR PL APO objective (Leica).

### Statistics

Statistical analyses of the results obtained in various groups were performed with Mann-Whitney U test. A p-value of <0.05 was considered to be statistically significant.

## Supporting Information

Figure S1 Gene expression of TLR and molecules of the TLR signaling cascade in HEK293 cells. (A) TLR gene expression of HEK293 cells only transfected with TLR (grey bars; n = 3) and HEK293 cells transfected with TLR and HLA-DR (black bars; n = 3). (B) Wild-type HEK293 cell lines obtained from the American Type Collection Culture (grey bars; n = 5) expressed comparable levels of molecules of the TLR signaling cascade genes as HLA-DR+ HEK293 cell lines (black bars; n = 5) obtained from Dr. J. Neefjes (Netherlands Cancer Institute). Error bars represent standard errors.
(1.05 MB TIF)

Figure S2 Gene expression of TLR, CD14, and molecules of the TLR signaling cascade in peritoneal macrophages of wild-type and MHC class II knock-out mice. Peritoneal macrophages of wild-type mice (black bars, n = 4) express comparable amounts of CD14, TLR2, TLR4, and molecules of the TLR signaling cascade genes as peritoneal macrophages of MHC class II knock-out mice (grey bars, n = 6). Error bars represent standard errors.
(0.33 MB TIF)
