# Supplementary figures and images for: MHC Class II Molecules Enhance Toll-Like Receptor Mediated Innate Immune Responses
Source: PLoS One. 2010 Jan 20;5(1):e8808. doi: 10.1371/journal.pone.0008808 (PMC2808354; doi:10.1371/journal.pone.0008808)

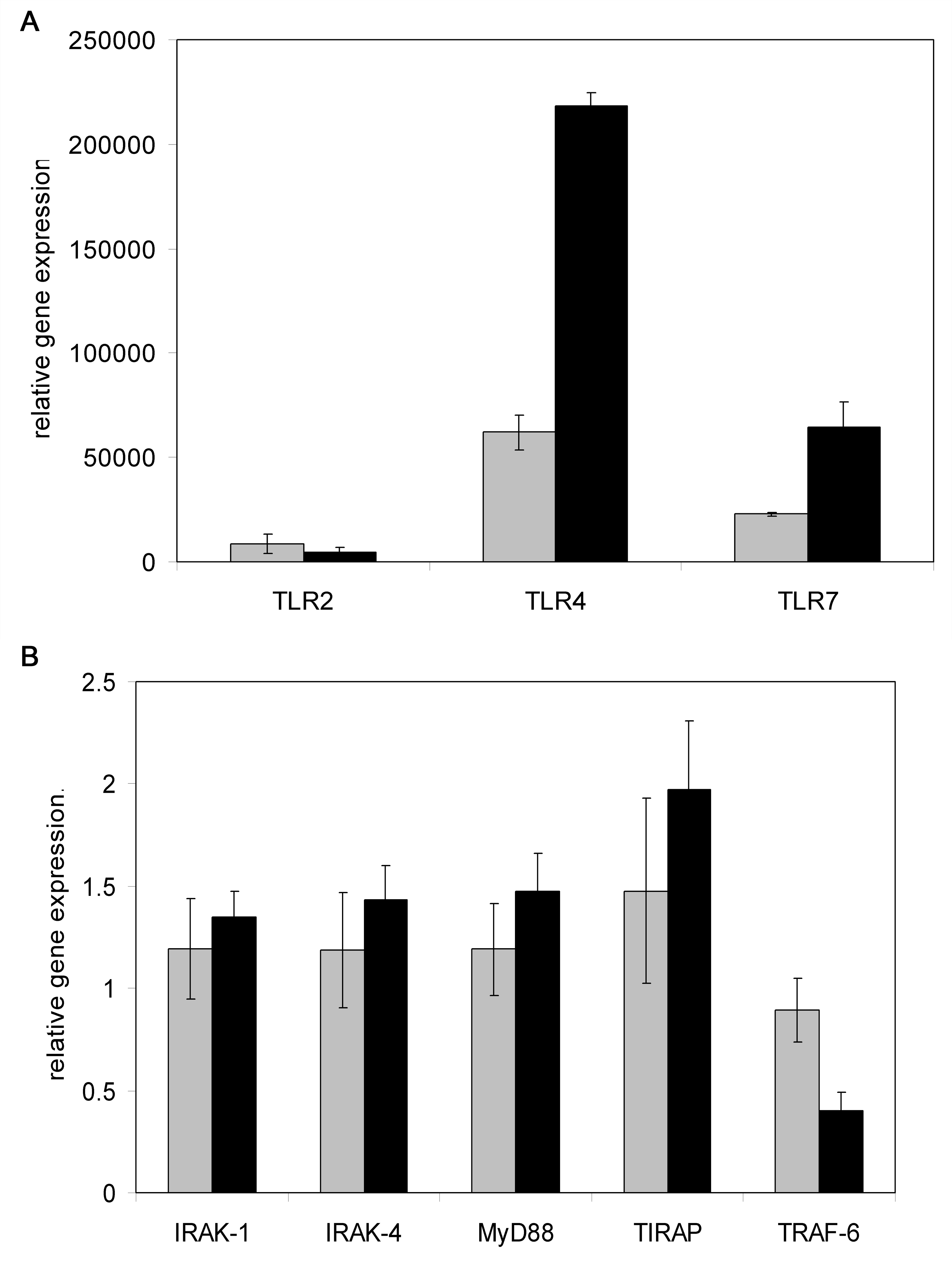

Supplement: Figure S1 — Gene expression of TLR and molecules of the TLR signaling cascade in HEK293 cells. (A) TLR gene expression of HEK293 cells only transfected with TLR (grey bars; n = 3) and HEK293 cells transfected with TLR and HLA-DR (black bars; n = 3). (B) Wild-type HEK293 cell lines obtained from the American Type Collection Culture (grey bars; n = 5) expressed comparable levels of molecules of the TLR signaling cascade genes as HLA-DR+ HEK293 cell lines (black bars; n = 5) obtained from Dr. J. Neefjes (Netherlands Cancer Institute). Error bars represent standard errors. (1.05 MB TIF) [file pone.0008808.s001.tif]

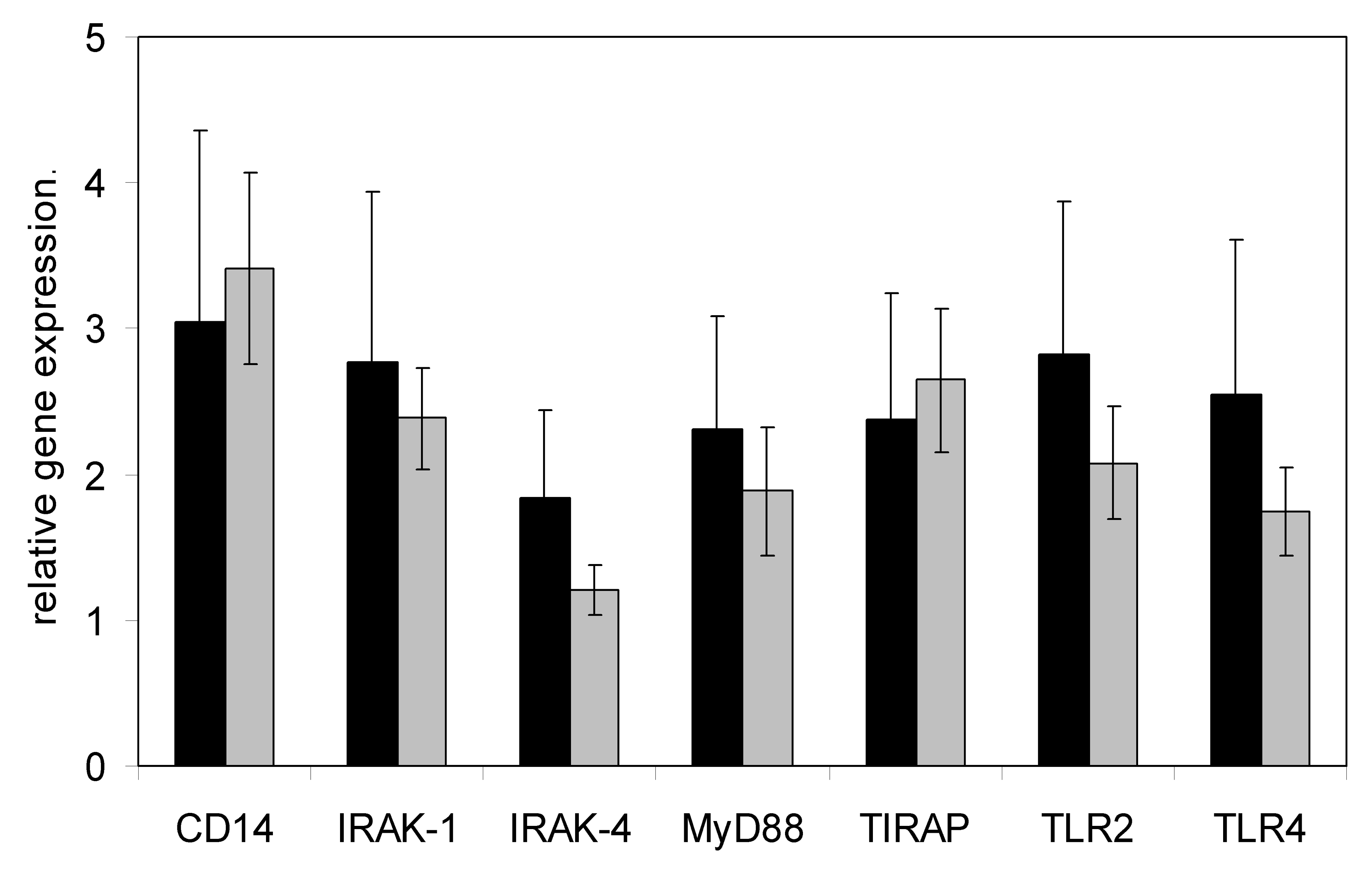

Supplement: Figure S2 — Gene expression of TLR, CD14, and molecules of the TLR signaling cascade in peritoneal macrophages of wild-type and MHC class II knock-out mice. Peritoneal macrophages of wild-type mice (black bars, n = 4) express comparable amounts of CD14, TLR2, TLR4, and molecules of the TLR signaling cascade genes as peritoneal macrophages of MHC class II knock-out mice (grey bars, n = 6). Error bars represent standard errors. (0.33 MB TIF) [file pone.0008808.s002.tif]
